# Pragmatic controlled trial to prevent childhood obesity in maternity and child health care clinics: pregnancy and infant weight outcomes (The VACOPP Study)

**DOI:** 10.1186/1471-2431-13-80

**Published:** 2013-05-20

**Authors:** Taina Mustila, Jani Raitanen, Päivi Keskinen, Antti Saari, Riitta Luoto

**Affiliations:** 1 Seinäjoki Central Hospital, Hanneksenrinne 7, 60220 Seinäjoki, Finland; 2 UKK Institute for Health Promotion,33501 Tampere, Finland; 3 Tampere School of Health Sciences, University of Tampere, Tampere, Finland; 4 Pediatric Research Centre, University of Tampere, 33014 Tampere, Finland; 5 Tampere University Hospital, 33521 Tampere, Finland; 6 Kuopio University Hospital, Kuopio, Finland; 7 University of Eastern Finland, Kuopio, Finland; 8 National Institute for Health and Welfare, 00271 Helsinki, Finland

**Keywords:** Childhood obesity, Gestational diabetes mellitus, Diet, Physical activity, Intervention, Prevention

## Abstract

**Background:**

According to current evidence, the prevention of obesity should start early in life. Even the prenatal environment may expose a child to unhealthy weight gain; maternal gestational diabetes is known to be among the prenatal risk factors conducive to obesity. Here we report the effects of antenatal dietary and physical activity counselling on pregnancy and infant weight gain outcomes.

**Methods:**

The study was a non-randomised controlled pragmatic trial aiming to prevent childhood obesity, the setting being municipal maternity health care clinics. The participants (n = 185) were mothers at risk of developing gestational diabetes mellitus and their offspring. The children of the intervention group mothers were born between 2009 and 2010, and children of the control group in 2008. The intervention started between 10–17 gestational weeks and consisted of individual counselling on diet and physical activity by a public health nurse, and two group counselling sessions by a dietician and a physiotherapist. The expectant mothers also received a written information leaflet to motivate them to breastfeed their offspring for at least 6 months. We report the proportion of mothers with pathological glucose tolerance at 26–28 weeks’ gestation, the mother’s gestational weight gain (GWG) and newborn anthropometry. Infant weight gain from 0 to 12 months of age was assessed as weight-for-length standard deviation scores (SDS) and mixed effect linear regression models.

**Results:**

Intervention group mothers had fewer pathological oral glucose tolerance test results (14.6% vs. 29.2%; 95% CI 8.9 to 23.0% vs. 20.8 to 39.4%; p-value 0.016) suggesting that the intervention improved gestational glucose tolerance. Mother’s GWG, newborn anthropometry or infant weight gain did not differ significantly between the groups.

**Conclusion:**

Since the intervention reduced the prevalence of gestational diabetes mellitus, it may have the potential to diminish obesity risk in offspring. However, results from earlier studies suggest that the possible effect on the offspring’s weight gain may manifest only later in childhood.

**Trial registration:**

Clinical Trials gov: NCT00970710

## Background

The increase in obesity prevalence in recent decades and the fact that obesity is difficult to reverse, even in childhood, has led to the conclusion that the prevention of obesity is the most effective way to combat this major health problem [1]. Obesity tends to originate in early life: nearly 11.3% of 2-year-old girls and 6.3% of boys of the same age in Finland are overweight or obese and, according to recently reported prevalence data, nearly 19% of 12–24 month-old children in the USA are obese [2,3]. The majority of obese pre-schoolers become obese schoolchildren and adults, which leads to an increased risk of cardiovascular diseases in adulthood [4,5].

Causes of obesity in pre-schoolers are multifactorial. Known modifiable risk factors in early life are mother’s obesity before pregnancy, as well as excessive weight gain, impaired glucose tolerance and smoking during pregnancy [6-11]. Moreover, the type of infant feeding, sleep duration and rapid weight gain during the first year of life are known risk factors for childhood obesity [12-18]. High birth weight and ponderal index (weight in kilograms divided by length in metres cubed, kg/m^3^) have been shown to have some effect on subsequent obesity risk, but evidence is weak [19,20]. Mother’s gestational diabetes (GDM) appears to increase the risk of obesity in offspring, even if the birth weight is normal [19,21].

Preventive efforts should start in early life. Pragmatic trials are needed to find an effective preventive programme that is applicable in existing health care settings [22]. Pregnant mothers and families with a preschool age child are often receptive to counselling on the health and well-being of their offspring. These families are easy to reach by primary health care. The offspring adopt dietary preferences during early life even via flavour in amniotic fluid and breast milk [23,24]. Dietary and physical activity habits are adopted during the preschool years [25,26]. To improve the cost-effectiveness of a programme that is carried out in a health care system, it should target families at risk of having obese offspring. One such risk group is the offspring of mothers at risk of developing GDM [19,21]. Mothers who are overweight or obese, those who have had gestational diabetes or a macrosomic newborn in a previous pregnancy, or those with an immediate family history of diabetes are considered to be at risk of GDM. These mothers often have a genetic predisposition to obesity and type −2 diabetes, which they may pass on to their offspring.

To the best of our knowledge there are no published intervention studies starting during the first trimester of pregnancy and aiming primarily at the prevention of overweight in the offspring. Only a few obesity prevention programmes targeting infancy have been reported, and they have mostly had short intervention and follow-up periods [27,28]. Some of these have shown a positive effect on children’s weight development [27-31]. Diet and physical exercise counselling targeting mothers during the infant’s first year seemed to result in slower weight gain in the offspring by the age of four years in a pilot study [29], while the same intervention during pregnancy did not significantly reduce the offspring’s weight gain [32]. Gillman et al. showed that treating mild GDM had no effect on the offspring’s weight status by the age of 4–5 years [33]. In the follow-up of the HAPO Study, glucose levels during pregnancy were not found to correlate significantly with offspring’s weight gain until two years of age [34].

The evidence from the obesity prevention programmes reported has shown that multifaceted intervention is more effective than targeting a single behaviour [35,36]. A recent meta-analysis of gestational interventions concluded that the evidence was low to very low for preventing gestational diabetes, but dietary and lifestyle interventions in pregnancy can reduce maternal GWG and improve outcomes for both mother and baby [37].

In this article we report the first results of an ongoing multifaceted controlled lifestyle intervention trial intended to prevent childhood obesity (The VACOPP, Vaasa Childhood Obesity Primary Prevention, Study) [38]. The study is being implemented in maternity and child health care clinics in the city of Vaasa in Western Finland. The intervention started during the first trimester of pregnancy and first targeted pregnant mothers at maternity health care clinics and then families until the offspring’s age of five years. In this paper we report the intermediate outcomes of the intervention given during pregnancy, such as the prevalence of GDM, mother’s GWG, newborn weight and infant weight gain in the groups.

## Methods

### Design and participants

The study was a non-randomised controlled clinical trial. All eight municipal maternity and 14 child health care clinics in the city of Vaasa in Western Finland participated in the recruitment and intervention. The intervention group mothers were recruited among the GDM risk group who were pregnant between February 2009 and April 2010; their offspring comprise the intervention target children. The control group was recruited among GDM risk group mothers and their offspring born in 2008 before the offspring had reached the age of one year.

The mothers who fulfilled the following criteria were considered to be at risk of GDM: body mass index (BMI) ≥25 kg/m^2^, macrosomic newborn (weight ≥4500 g) or GDM in any previous pregnancy, an immediate family history of diabetes and/or age ≥40 years. In Finland this group of mothers is routinely offered an oral glucose tolerance test (OGTT) when 26–28 weeks pregnant. The children of these mothers were the primary target of the intervention. Mothers with multiple pregnancy, who were unable to speak Finnish, with substance abuse or severe psychiatric problems were excluded from the study.

Public health nurses (PHN) recruited the intervention group in maternity health care clinics at the first personal contact not later than 12th gestational week, and provided the expectant mothers with written information about the research and consent forms. The children of the intervention group mothers were born in 2009 or 2010. The control group was recruited among mothers who had undergone OGTT in mid-pregnancy because of a risk of GDM according to the above mentioned criteria. The control mothers gave birth in 2008 (January – December). The control group mothers were identified from the laboratory register for maternity care. The research nurses contacted them by telephone in 2009 requesting their permission to send them written information on the research and consent forms. All mothers recruited were offered an opportunity to address questions about the trial by telephone or e-mail to either the research nurse or the researchers. Informed written consent was provided by all participant mothers prior to the baseline assessments.

In our study power calculations were not primarily calculated, since the project was intended to pilot an approach of pragmatic design to the early prevention of childhood obesity. Power calculations based on mean BMI z-score in the control group would probably be inaccurate [39]. Ethical approval for the study was granted by the Ethics Committee of Vaasa Hospital District.

### Intervention

The ante-natal intervention consisted of two group counselling sessions, during the first and second trimesters of pregnancy. Sessions lasting 1.5 hours were given by a trained physiotherapist and a dietician working in public health care. Counselling on diet especially emphasised the recommended consumption of fibre, energy content, quality of carbohydrates and fat in the diet [40]. During counselling sessions the expectant mothers received information on suitable and sufficient exercise during pregnancy. They also participated in a brief session of muscle tone exercise that could be repeated at home. Mothers were advised to exercise for at least 2.5 hours/week (until at least slightly out of breath) and to do muscle tone training twice a week [41]. Information about the effect of a healthy diet, exercise and appropriate weight gain during pregnancy on the risk of contracting GDM, offspring’s perinatal problems and obesity in the offspring was given to the participant mothers. Written educational material on a healthy diet and physical activity during pregnancy was distributed during the sessions. These counselling sessions followed a structured schema and thus all mothers received the same information from the dietician and the physiotherapist.

During the 13 routine visits to the maternity health care clinics starting from tenth week of pregnancy, the PHNs briefly recapped the counselling information provided during the group sessions. According to the nature of the pragmatic trials the counselling offered by PHNs may have varied in content and the time spent on it. Pragmatic trial counselling should also be tailored according to the client’s needs. At the first home visit to the mother and baby the PHN gave mother an information leaflet recommending breastfeeding until six months of child’s age to promote appropriate weight gain in the infant. The intervention is described in more detail in the protocol article [38].

### Outcome measures

The secondary outcomes of the VACOPP Study until child’s age of one year are described in this report. The primary outcome will be BMI and proportion of overweight or obese children at the age of six years [38]. Maternal outcomes were self-reported duration of exercise (until at least slightly out of breath) during the second and the third trimesters of pregnancy, OGTT results at 26–28 weeks’ gestation, and GWG until 37 weeks of pregnancy. The GWG was assessed at 37 weeks’ gestation to ascertain the most recent weight in the maternity clinic as comprehensively as possible for all mothers, and also because later in pregnancy weight may be greatly affected by swelling. The OGTT was performed with a 75 gram glucose load. The plasma glucose values were analysed from capillary plasma samples at Vaasa Central Hospital laboratory. The following OGTT cut-off levels were used for capillary plasma glucose: 0 h ≥ 5.3 or 1 h ≥ 11.0 or 2 h ≥ 9.6 mmol/l [42]. The OGTT was considered abnormal if one of those values exceeded the cut-off level. Neonatal outcomes were the proportion of non-complicated vaginal deliveries, birth weight, newborn ponderal index and large- or small-for-gestational age status. Infant outcomes reported here are the duration of exclusive breastfeeding, differences in weight-for-length SDSs and changes in weight-for-length SDSs at 0, 4, 6 and 12 months of age. We also report the absolute BMI differences in the groups, and the proportions of overweight and obese infants (overweight reaching or exceeding +10% and obese reaching or exceeding + 20% curves for weight-for-length above the mean weight-for-height of healthy Finnish children) according to the new Finnish growth reference [43]. These weight-for-length/height curves with percentual deviation of the mean are widely used in Finnish health care. The educational level of parents is defined as follows: “low” corresponds to education up to vocational school; “medium” indicates a polytechnic degree and “high” a university degree.

### Data collection

Mothers’ weight was measured to the nearest 0.1 kg in light clothing on a standard electronic scale by maternity health care clinic PHNs. Maternal BP (blood pressure) was measured by the same PHN using an automated BP monitor (Omron M6) under standard conditions with two repeated measurements. Mother’s weight gain, BP, mother’s own estimate on her weekly physical exercise during pregnancy, and the results of 2-hour 75 gram OGTT at 26–28 weeks of pregnancy were recorded in the questionnaires. The questionnaires were completed partly by the PHN and partly by the mothers during the first, second and third trimesters of pregnancy during the intervention group mothers’ visits to maternity health care. The PHN measured and wrote down the intervention group physical and laboratory measures for the questionnaires. Newborn anthropometry was measured at the hospital by a hospital nurse immediately after delivery and the study questionnaires were completed by the PHN at the first visit to maternity care after delivery.

The control group’s measures during pregnancy were entered in the questionnaires by the mothers themselves 1–12 months after the end of pregnancy in 2008. The control mothers transferred data concerning physical measures and OGTT results to the study questionnaires from their maternity cards, which were filled in by a PHN during their pregnancy. The researcher was able to check the OGTT results from the laboratory register if necessary. Fathers’ and grandparents’ anthropometry, possible diabetes diagnose and educational levels were reported by the participating mothers in both groups.

Child health care clinic PHNs weighed and measured the infants at routine visits at 4, 6 and 12 months. The infant’s weight was measured to the nearest 0.01 kg without clothing on a standard electronic scale. Infants’ length was measured in recumbent position to the nearest millimetre with a standard stadiometer. Both intervention and control group infant anthropometric measures were completed on the questionnaires by the PHN at the one-year visit to the child health care clinic. Long-term illnesses affecting growth (e.g. severe food allergies) and duration of exclusive breastfeeding (months) were likewise recorded in this questionnaire.

### Statistical methods

Characteristics of the study participants are described using means or frequencies and 95% confidence intervals (Tables 1, 2 and 3). Corresponding 95% confidence intervals (CI) for continuous variables were calculated using formula mean ± (1.96 * standard error of the mean) and for categorical variables using the Wilson score method without continuity according to Newcombe [44]. Differences between groups were evaluated using Student’s *t*-test or Mann–Whitney *U*-test for normally or non-normally distributed continuous variables. Normality was evaluated with the Kolmogorov-Smirnov test. Categorical variables were tested using the chi-square test or Fisher’s exact test. The child’s weight gain was analysed using weight and length converted to weight-for-length and their SDSs (z-scores) according to the recently updated Finnish growth reference [43]. To investigate the effect of the intervention on child’s weight, the outcome variable was the child’s weight-for-length z-score at 0, 4, 6 and 12 months of age. In order to take into account the within-child correlation between repeated measures, we used a multilevel mixed-effects linear regression models to analyse the association of the weight-for-length z-score over time by group (intervention/control). The model included a variable (group) to indicate the difference between groups at baseline and a variable (time) to indicate the changes of weight-for-length z-scores over time. The difference in the change in z-scores across the intervention between the two groups was tested using an interaction term between group and time, which can be viewed as the intervention effect. In addition, we added potential confounding variables to the model: mother’s education, number of pregnancies, smoking status during pregnancy, pre-pregnancy BMI, gender of the child and target height. None of these variables were significant, thus the final model only includes the three factors mentioned above. The goodness-of-fit of the model was evaluated by normal probability and residual plots and also tested by the normality of the residuals (Kolmogorov-Smirnov test). All analyses were performed using STATA software (version 12.0 for Windows), StataCorp LP, Texas, USA.

**Table 1:** Baseline characteristics of the trial groups (mean or frequency and 95% CI)

	**Intervention**	**Control**	**p-value**	**Missing (n in groups)**
N	96	89		
Age of mother before pregnancy (years)	30.9 (29.7 to 32.0)	30.1 (29.0 to 31.2)	0.37 ^a^	-
Mother’s education			0.82 ^c^	-
Low	32.3% (23.8% to 42.2%)	28.1% (19.8% to 38.2%)		
Medium	43.8% (34.3% to 53.8%)	46.1% (36.1% to 56.4%)		
High	24.0% (16.6% to 33.4%)	25.8% (17.8% to 35.8%)		
Father’s education			0.27 ^c^	1, 4
Low	34.7% (25.9% to 44.7%)	35.3% (26.0% to 45.9%)		
Medium	36.8% (27.8% to 46.8%)	45.9% (35.7% to 56.4%)		
High	28.4% (20.3% to 38.2%)	18.8% (11.9% to 28.4%)		
Mother’s pre-pregnancy BMI (kg/m^2^)	27.5 (26.6 to 28.5)	26.6 (25.7 to 27.4)	0.15 ^a^	-
Proportion of obese mothers (BMI ≥ 30 kg/m^2^)	26.0% (18.3% to 35.6%)	19.1% (12.3% to 28.5%)	0.26 ^c^	-
Father’s BMI (kg/m^2^)	27.3 (26.5 to 28.1)	27.1 (26.2 to 28.0)	0.86 ^b^	2, 6
Proportion of obese fathers (BMI ≥ 30 kg/m^2^)	20.2% (13.3% to 29.4%)	16.9% (10.3% to 26.4%)	0.57 ^c^	2, 6
Mother, Type −2 Diabetes	0.0% (0.0% to 3.9%)	1.1% (0.2% to 6.1%)	0.48 ^d^	1, 0
Father, Type −2 Diabetes	1.1% (0.2% to 5.8%)	1.1% (0.2% to 6.2%)	1.00 ^d^	2, 2
Proportion of obese grandparent (BMI ≥ 30 kg/m^2^)	56.8% (46.4% to 66.7%)	63.1% (52.4% to 72.6%)	0.40 ^c^	8, 5
Proportion of a grandparent having type −2 Diabetes	39.1% (29.8% to 49.3%)	43.2% (33.0% to 54.1%)	0.59 ^c^	4, 8
Parity			0.24 ^c^	-
Primiparous	57.3% (47.3% to 66.7%)	43.8% (35.0% to 55.3%)		
Second pregnancy	26.0% (18.3% to 35.6%)	32.6% (23.7% to 42.9%)		
At least third pregnancy	16.7% (10.5% to 25.4%)	23.6% (15.0% to 32.2%)		
History of newborn >4500 g	2.1% (0.6% to 7.4%)	3.4% (1.2% to 9.4%)	0.60 ^c^	1, 0
Mother smoking during pregnancy	5.2% (2.2% to 11.6%)	11.2% (6.2% to 19.5%)	0.13 ^c^	-
Mother’s physical activity (hours/week) during first trimester of pregnancy (before intervention)*	4.5 (3.9 to 5.1)	4.7 (3.8 to 5.6)	0.41 ^b^	2, 5

^a^ Independent Samples *T*-test, ^b^ Mann–Whitney *U*-test, ^c^ Chi-Square Test, ^d^ Fisher’s Exact Test.

BMI = body mass index; CI, confidence interval; *level of at least slightly out of breath.

**Table 2:** Secondary maternal and neonatal outcomes in the trial groups (mean or frequency and 95% CI)

	**Intervention**	**Control**	**p value**	**Missing (n in groups)**
N	96	89		
*Maternal*				
First trimester				
Systolic blood pressure (mmHg)	119.1 (116.9 to 121.2)	116.5 (114.3 to 118.7)	0.10 ^a^	4, 5
Diastolic blood pressure (mmHg)	73.9 (72.4 to 75.4)	72.1 (70.0 to 74.1)	0.14 ^a^	4, 5
Second trimester				
Systolic blood pressure (mmHg)	116.8 (114.7 to 119.0)	117.7 (115.4 to 119.9)	0.59 ^a^	2, 6
Diastolic blood pressure (mmHg)	71.7 (70.1 to 73.3)	70.5 (68.5 to 72.5)	0.33 ^a^	2, 6
Physical exercise (h/week)*	4.2 (3.6 to 4.7)	4.5 (3.6 to 5.4)	0.62 ^b^	2, 5
OGTT (Gestational weeks 26–28)				
Fasting-0 h (mmol/l)	4.8 (4.7 to 4.8)	4.9 (4.8 to 5.0)	0.12 ^b^	-
1 h (mmol/l)	8.7 (8.4 to 9.0)	9.0 (8.7 to 9.4)	0.21 ^a^	-
2 h (mmol/l)	6.8 (6.6 to 7.1)	6.9 (6.6 to 7.1)	0.77 ^a^	-
Pathological OGTT result (cP)	14.6% (8.9% to 23.0%)	29.2% (20.8% to 39.4%)	0.016^c^	
Third trimester				
Systolic blood pressure (mmHG)	122.4 (120.1 to 124.6)	122.5 (120.0 to 125.0)	0.79^b^	3, 4
Diastolic blood pressure (mmHG)	77.8 (76.1 to 79.5)	75.2 (73.2 to 77.3)	0.052^a^	3, 4
Physical exercise (h/week)*	3.4 (3.0 to 3.8)	3.2 (2.5 to 3.9)	0.11^b^	4, 4
Gestational weight gain until 37 gw (kg)	11.4 (10.4 to 12.5)	12.7 (11.5 to 14.0)	0.11^a^	2, 0
Min – Max	−4.9 to 27.2	−1.0 to 34.7		
*Neonatal*				
Non-complicated vaginal delivery	77.1% (67.7% to 84.4%)	75.3% (65.4% to 83.1%)	0.77^c^	-
Gestational age at birth	39.8 (39.4 to 40.1)	39.4 (39.2 to 39.7)	0.084^b^	-
Sex of the newborn (boy)	51.0% (41.2% to 60.8%)	50.6% (40.4% to 60.7%)	0.95^c^	-
Birth weight (grams)	3509 (3404 to 3615)	3507 (3417 to 3596)	0.97^a^	-
Ponderal index (weight, kg/length, m^3^)	27.4 (26.9 to 27.9)	27.5 (27.0 to 27.9)	0.89^a^	-
Large for gestational age	7.3% (3.6% to 14.3%)	5.6% (2.4% to 12.5%)	0.64^c^	-
Small for gestational age	13.5% (8.1% to 21.8%)	6.7% (3.1% to 13.9%)	0.13^c^	-
Exclusive breastfeeding (months)	3.0 (2.5 to 3.4)	2.8 (2.3 to 3.2)	0.52^b^	8, 0

^a^Independent Samples *T*-test, ^**b**^Mann-Whitney *U*-test, ^c^Chi-Square Test, ^d^Fisher’s Exact Test; OGTT, oral glucose tolerance test (75 g glucose load, 2-hour); cP = capillary plasma glucose; CI, confidence interval; *level of at least slightly out of breath; gw, gestational weeks.

**Table 3:** Anthropometric data in study groups during child’s first year (mean ± sd or frequency and %)

	**Intervention**	**Control**	**p-value**	**Missing (n/group)**
N	96	89		
**Weight-for-length SDS**				
0 months	−0.08 ± 0.96	−0.07 ± 0.93	0.94^a^	
4 months	0.05 ± 0.99	0.17 ± 1.10	0.46^a^	
6 months	0.13 ± 1.02	0.20 ± 1.18	0.65^a^	
12 months	0.09 ± 1.06	0.06 ± 1.11	0.85^a^	3, 0
**Change in weight-for-length SDS**				
0 to 4 months	0.13 ± 1.17	0.24 ± 1.28	0.56^b^	
0 to 6 months	0.21 ± 1.14	0.27 ± 1.38	0.74^b^	
0 to 12 months	0.16 ± 1.20	0.14 ± 1.39	0.89^b^	3, 0
4 to 12 months	0.05 ± 0.90	−0.10 ± 0.74	0.21^b^	3, 0
6 to 12 months	−0.02 ± 0.74	−0.14 ± 0.66	0.28^b^	3, 0
**Change in weight-for-length SDS ≥ 0.67**				
0 to 4 months	32 (33.3%)	31 (34.8%)	0.83^c^	
0 to 6 months	33 (34.4%)	36 (40.4%)	0.39^c^	
0 to 12 months	32 (34.4%)	31 (34.8%)	0.95^c^	3, 0
4 to 12 months	20 (21.5%)	13 (14.6%)	0.23^c^	3, 0
6 to 12 months	13 (14.0%)	11 (12.4%)	0.75^c^	3, 0
**Change in weight-for-length SDS ≤ − 0.67**				
0 to 4 months	25 (26.0%)	22 (24.7%)	0.84^c^	
0 to 6 months	21 (21.9%)	23 (25.8%)	0.53^c^	
0 to 12 months	23 (24.7%)	23 (25.8%)	0.86^c^	3, 0
4 to 12 months	23 (24.7%)	17 (19.1%)	0.36^c^	3, 0
6 to 12 months	21 (22.6%)	16 (18.0%)	0.44^c^	3, 0
**Weight-for-length ≥ +10% **^e^				
0 months	9 (9.4%)	10 (11.2%)	0.68^c^	
4 months	15 (15.6%)	18 (20.2%)	0.41^c^	
6 months	15 (15.6%)	22 (24.7%)	0.12^c^	
12 months	16 (17.2%)	18 (20.2%)	0.60^c^	3, 0
**Weight-for-length > +20% **^f^				
0 months	1 (1.0%)	1 (1.1%)	1.00^d^	
4 months	0 (0.0%)	4 (4.5%)	0.052^d^	
6 months	4 (4.2%)	5 (5.6%)	0.74^d^	
12 months	3 (3.2%)	1 (1.1%)	0.62^d^	3, 0
**Body mass index** (kg/m2)				
0 months	13.8 ± 1.3	13.8 ± 1.1	0.93^a^	
4 months	17.0 ± 1.4	17.2 ± 1.6	0.32^a^	
6 months	17.4 ± 1.5	17.6 ± 1.7	0.58^a^	
12 months	17.2 ± 1.4	17.2 ± 1.6	0.89^a^	3, 0

^a^Independent Samples *T*-test, ^b^Linear regression analysis, unadjusted, ^c^Chi-Square Test, ^d^Fisher’s Exact Test.

^e^assessed as overweight, ^f^assessed as obese; SDS, standard deviation score.

## Results

**Figure 1:**
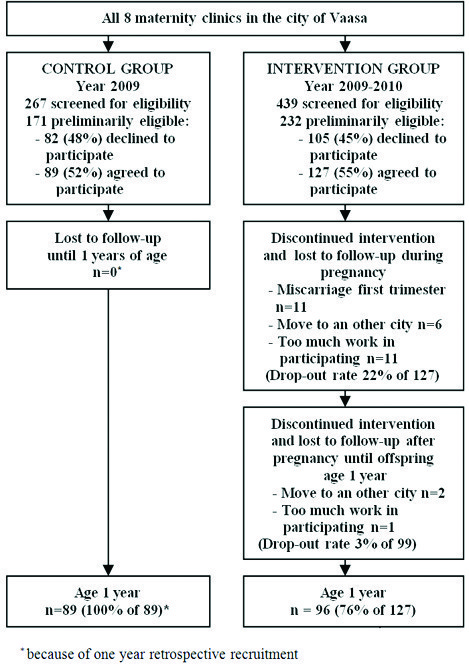
Flow chart of the study. *Because of one year retrospective recruitment.

The study flow is described in Figure 1. Roughly 700 hundred women per year give birth in the city of Vaasa. According to the birth registry for 2009 about one third of women in Finland are at least overweight before pregnancy contributing to the risk group of GDM. We analysed baseline characteristics that might interfere with offspring’s weight development and found no statistically significant differences between the groups (Table 1). Of the intervention group mothers 84/96 (87.5%) participated in the first trimester counselling sessions held by a dietician and a physiotherapist, and 57/96 (59.4%) in the corresponding session during the second trimester. The participation rate with regard to the PHN counselling was close to 100% since the counselling was held during routine visits to the maternity health care clinics.

No statistically significant differences were found in the weekly duration of pregnant mothers’ exercise during the second and the third trimesters of pregnancy or in GWG until 37 gestational weeks (p-value 0.11) (Table 2). We also analysed the weight gain in groups in relation to pre-pregnancy BMI according to IOM: mothers keeping within the recommended total weight gain range, mothers below it and mothers exceeding the range [45]. The proportion of mothers exceeding the recommended range in the intervention group was 43.6% and in the control group 47.2%, but the difference was not statistically significant (p-value 0.83). The differences in the proportions of mothers keeping within or below the recommendations were also not significant between the groups (data not shown). We further analysed the association of exceeding GWG recommendations and pathological OGTT result and did not find it to be statistically significant (p-value 0.097, data not shown).

The control group mothers had a significantly higher proportion of abnormal OGTT results than the intervention group (29.2% vs. 14.6%, p-value 0.016). The proportion of mothers having a non-complicated delivery was similar in both groups. Nor was there significant difference in the newborn anthropometry (birth weight, ponderal index, large-for-gestational age status, or small-for-gestational age status). No significant differences were found between the groups for the mothers’ pregnancy BP level, the proportion of mothers gaining less weight than recommended, the duration of pregnancies or the small-for-age status of newborns. Nor was there any significant difference in the proportions of infants with slow weight gain. We interpreted the above-mentioned results as indications of the safety of the intervention.

Duration of exclusive breastfeeding did not differ statistically significantly between the groups (p-value 0.52) (Table 2). In addition, no statistically significant differences were found in length-for-age SDS, weight-for-age SDS, or weight-for-length SDS at 0, 4, 6 and 12 months of age between the groups (all data not shown). Proportions (expressed as percentage value deviation from the mean weight-for-length value according to Finnish definition of preschool-age overweight and obesity) of overweight (≥ + 10% weight-for-length) or obese (≥ + 20% weight-for-length) infants at ages 4, 6 and 12 months were not significantly different between the groups, although a slight tendency for the control group to have a higher proportion of overweight infants was seen (Table 3) [43]. There were no differences in weight gain velocity assessed as change in weight-for-length ≥ 0.67 SDS or ≤ −0.67 SDS between the groups. Because of the lack of a Finnish age- and gender-adjusted BMI reference for children under two years of age, we could not analyse BMI SDS. Absolute BMI was similar in the groups at the ages of 0, 4, 6 and 12 months (Table 3). Mixed effect linear regression models included group and age of the child and interactions between group and age of the child. Adding gender and the target height of the child, mother’s pre-pregnancy BMI, smoking status during pregnancy, number of pregnancies and mother’s educational level to the models did not induce significant differences to the results, and they were not included in the results reported. According to a mixed effect linear regression model, the z-score slopes did not differ significantly between the intervention and control groups (p-value 0.71) (Table 4).

**Table 4:** Estimates and 95% confidence intervals for weight-for-length SDS from multilevel mixed-effects linear regression model

**Weight-for-length SDS from 0 to 12 months of age**	**Coefficient**	**95% CI**	**p-value**
Group (intervention/control)	−0.71	−0.31 to 0.16	0.56
Age in months	−0.06	0.02 to 0.01	0.002
Age in months ^2^	−0.004	−0.007 to −0.002	0.002
Group * Age	−0.006	−0.023 to 0.034	0.71

SDS, standard deviation score; non-linear relationship between SDS and age of the child was modelled using polynomial age in months ^2^; Group * Age = interaction between age of the child and the group.

We performed post study power analysis for the differences in infants’ weight gain and found that the groups should include 250 children when multilevel mixed-effects linear regression model is used to analyse the association of the weight-for-length z-score over time by group.

## Discussion

The results reported here suggest that the intervention in this trial may have the potential to improve glucose tolerance in pregnant mothers. According to earlier studies a lower gestational glucose level can have a positive long-term effect in reducing the child’s risk of obesity and type −2 diabetes [10,11,21]. The intervention did not have a significant effect on mother’s weight gain during pregnancy, although a slight tendency towards lower weight gain was seen among the intervention group mothers. No significant differences were found in the proportions of non-complicated deliveries, offspring’s birth weight or ponderal index. Also, the duration of exclusive breastfeeding was similar in both groups. Offspring growth during the first year was not statistically significantly different between the groups, but a slight tendency was noted for the control group to have a higher proportion of overweight offspring during the first year.

There is evidence of a favourable effect of lifestyle counselling during pregnancy on mother’s diet, glucose tolerance and foetal growth. Luoto et al. showed that counselling on dietary and physical activity during pregnancy was effective in reducing the proportion of large-for-age newborns [46]. They also showed that gestational intervention had a beneficial effect on several dietary aims, but only a non-significant effect on the increase in physical activity [46]. Barakat et al. recently reported an improvement in glucose tolerance in their physical activity intervention study during pregnancy [47]. We measured the mother’s physical activity by self-reports, and no significant differences between the groups were found. The control group’s physical activity was elicited and recorded 1–12 months after the pregnancy, therefore, a recall bias cannot be excluded. Recall bias may also affect the intervention group mothers’ estimates of exercise taken, because the data was gathered only once every trimester of pregnancy. Self-reports are also only rough estimates of the time spent being physically active.

The incidence of GDM in Finland is estimated to be close 11% according to the Medical Birth Register for 2004–2006 [48]. A fourth of overweight pregnant women have been estimated to get GDM. In the study by Luoto et al. a third of women in comparable risk group as ours got GDM [46]. The lower proportion of pathological OGTT results at 26–28 gestational weeks in the intervention group could be attributable to dietary changes. Since our main and secondary outcomes are the offspring’s measures, we did not keep any dietary records during pregnancy, but only started to gather these during the toddler years. An increase in fibre-rich food intake, which was one of the aims of the counselling, has the potential to improve glucose tolerance. Differences in energy consumptions between the groups seems more unlikely since there were only indicative differences between weight gain during pregnancy and no significant differences in self-reported physical activity. The first group intervention was offered not later than at 20 gestational weeks’ and the PHNs gave intensive counselling at mothers’ routine visits to maternity clinics beginning from 10 to 12 gestational weeks, making it possible that the intervention may have had an ameliorating effect on the OGTT results. The mothers were told that their lifestyle during pregnancy could have significant effects on the outcomes of the pregnancy and on their newborns, and also on the offspring’s weight development. We believe that this knowledge may have motivated the intervention mothers to make healthy dietary changes during pregnancy.

Mothers with abnormal OGTT results suggesting GDM and thereafter also abnormal plasma glucose values in self-monitoring are referred to the central hospital for further assessment and treatment. All mothers with GDM are given dietary advice contributing to better glucose balance and a glucose meter to monitor their glucose values at home. Insulin treatment is initiated if the target glucose balance is not achieved by these means. The effective treatment of GDM may have had an impact on the outcomes we measured: GWG, type of delivery and newborn weight. This could at least in part explain why there were no significant differences in these measures despite the higher prevalence of GDM among the control group mothers. There were no significant differences in the newborns’ birth weights, but it has been shown that the risk of giving birth to a macrosomic baby is related to mother’s BMI before pregnancy, when her GDM is well controlled [49]. Despite the lack of statistical significance, the somewhat lower gestational weight gain until 37 weeks of gestation according to confidence intervals, may be a result of the intervention and partly explain the lower incidence of GDM in the intervention group. It is also possible that the higher prevalence of GDM in the control group is a biased result from the insufficient power of the sample, type 1 error or a chance.

The offspring’s weight-for-length was analysed and adjusted with the recently updated Finnish growth reference to obtain the SDS [43]. Weight gain velocity was analysed by assessing the proportions of infants whose change in weight-for-length was at least 0.67 SDS during their first 12 months. A weight-for-age difference of >0.67 SD has been defined as a clinically relevant rapid weight gain in infancy associated with a risk of obesity later in childhood [14]. Weight gain during infancy was analysed with mixed effect models allowing for a difference between groups at baseline, changes over time and intervention effects. No significant differences were found between the intervention and control group offspring’s weight gain during the first year of life. The intervention did not result in a longer duration of exclusive breastfeeding, which may result from the very light intervention in this issue. Improvements in foetal conditions such as mothers’ lower glucose level during pregnancy have been shown to have no positive effect on the offspring’s weight gain until the toddler years [9,10,19]. These above mentioned facts could explain why no differences in infant weight gains were seen despite the differences in mothers’ glucose tolerance in mid-pregnancy.

The total drop-out rate among the intervention group during pregnancy was 22% (Figure 1). The most common reasons for dropping-out were an early miscarriage or the mother finding the study intervention too taxing. The miscarriages occurred during the first trimester of the pregnancies (except for one registered in the 20th gestational week), excluding the effect of the intervention on miscarriage rate. High drop-out rate is usual in lifestyle interventions and in that regard the drop-out rate in our study during pregnancy is moderate. It is possible that the mothers who were most motivated to make lifestyle changes and at the lowest risk for GDM were the ones who continued in the intervention. This could have had an impact on GDM prevalence results in the groups.

The study groups were comparable at baseline as characteristics possibly interfering in the offspring’s risk of obesity showed no statistically significant differences between the groups. However, we were not able to obtain reliable data on the mothers’ possible previous GDM. We targeted a group of mothers at risk of developing GDM, thus making the possibility of the intervention effect higher. The intervention was started during foetal and infant life, which are the periods known to be risk periods for the future development of obesity.

Our study has several limitations. It is not randomised and the control group was up to one year retrospective concerning outcomes in pregnancy, which could cause some bias in the results. The public health physiotherapist, dietician and maternity clinic PHNs only cared for the intervention group mothers, and the control group mothers’ and infants’ data was gathered when the control offspring was one year of age, that is one year before intervention was started, removing a possible Hawthorn effect on the control group.

The participation rate in the group counselling sessions during pregnancy was good, although it was lower in the second session. Almost 98% of the mothers in Finland avail themselves of the planned routine visits to municipal maternity health care. Thus, the risk mothers are more effectively reached if the intensified counselling is arranged in connection with these routine visits, which was partly the case in our study.

Some of the public health nurses particularly felt that the recruitment of the intervention group and the paperwork of the study were burdensome, mostly because of their hectic pace of work. This may have attenuated the success of recruiting in some maternity clinics. The motivation of the PHNs to perform intensive counselling may have varied, making the intervention uneven. However, this is common in real life implementations and the results are in this respect comparable to these settings. Implementation in real-life practice has good potential to be a sustainable part of municipal health care if proved to be effective. The maternity and child health care clinics have a good opportunity to reach the risk population for childhood obesity during a life phase when the families are motivated to make changes in their behaviour in order to promote their offspring’s health.

## Conclusions

Obesity with its great health and economic burden challenges society to initiate preventive actions. The most natural setting in primary health care for preventive interventions is maternity and child health care clinics, as this reaches the beginning of next generation. To find effective prevention programmes pragmatic trials in the real-life setting are needed. Our study appeared to improve glucose tolerance during pregnancy, suggesting its potential to have a positive effect on offspring weight gain. We failed to find any effect on newborn birth weight or infant weight gain, but research has shown that an adverse effect of gestational diabetes on the offspring’s weight gain tends to develop only later in childhood. Several ongoing early life intervention studies to prevent childhood obesity will provide more evidence on the feasibility of programmes for implementing in practice, including our intervention and follow-up continuing in child health care centres during preschool years.

## Abbreviations

GWG: Gestational weight gain; SDS: Standard deviation score; GDM: Gestational diabetes mellitus; BMI: Body mass index; OGTT: Oral glucose tolerance test; PHN: Public health nurse; BP: Blood pressure; CI: Confidence interval.

## Competing interests

The authors declare that they have no competing interests.

## Authors’ contributions

TM and PK contributed to the conception and design of the study. TM coded the data. TM, RL and PK participated in drafting and revising the manuscript. JR and TM performed the statistical analysis. AS produced the weight-for-length statistics and participated in the interpretation of these results. All authors had full access to all of the data (including statistical reports and tables) in the study and can take responsibility for the integrity of the data and the accuracy of the data analysis. All authors read and approved the final version of the manuscript.

## Pre-publication history

The pre-publication history for this paper can be accessed here:

http://www.biomedcentral.com/1471-2431/13/80/prepub
